# Assessing Excess Mortality of Baby Boomers from the COVID-19 Pandemic: Taiwan Omicron-naïve Cohort

**DOI:** 10.1007/s44197-024-00262-0

**Published:** 2024-06-20

**Authors:** Ting-Yu Lin, Chen-Yang Hsu, Amy Ming-Fang Yen, Sam Li-Sheng Chen, Tony Hsiu-Hsi Chen

**Affiliations:** 1 Institute of Epidemiology and Preventive Medicine, College of Public Health, National Taiwan University, Room 533, No. 17, Xu-Zhou Road, Taipei, 100 Taiwan; 2 Department of Emergency, Dachung Hospital, Miaoli, Taiwan; 3 School of Oral Hygiene, College of Oral Medicine, Taipei Medical University, Taipei, Taiwan

**Keywords:** COVID-19, Excess mortality, Baby-boomer

## Abstract

**Background:**

Asia’s elderly Baby Boomer demographic (born between 1946 and 1964) faced a huge problem during the COVID-19 pandemic due to increased all-cause mortality. We aimed to provide a unique Taiwan situation regarding the impact of Baby Boomers on excess mortalities from all causes relative to non-Baby Boomers throughout distinct times of SARS-CoV-2 mutations during the COVID-19 pandemic.

**Methods:**

We used the Poisson time series design with a Bayesian directed acyclic graphic approach to build the background mortality prior to the COVID-19 pandemic between 2015 and 2019. It was then used for predicting the expected all-cause deaths compared to the reported figures during the COVID-19 pandemic period based on Taiwan residents, an Omicron-naïve cohort.

**Results:**

Baby Boomers experienced a 2% negative excess mortality in 2020 (Wuhan/D614G) and a 4% excess mortality in 2021 (Alpha/Delta) with a rising background mortality trend whereas non-Baby Boomers showed the corresponding figures of 4% negative excess and 1% excess with a stable trend. Baby Boomer and non-Baby Boomer excess mortality soared to 9% (95% CI: 7-10%) and 10% (95% CI: 9-11%), respectively, during the epidemic Omicron period from January to June 2022. Surprisingly, Baby Boomers aged 58–76 experienced the same 9% excess mortality as non-Baby Boomers aged 77 and beyond. Non-COVID-19 deaths were more prevalent among Baby Boomers than non-Baby Boomers (33% vs. 29%).

**Conclusion:**

Baby Boomers were more likely to die from COVID-19 in early pandemic and had more non-COVID-19 deaths in late pandemic than older non-Baby Boomers demonstrated in Taiwan Omicron-naïve cohort. For this vulnerable population, adequate access to medical care and medical capacity require more consideration.

**Supplementary Information:**

The online version contains supplementary material available at 10.1007/s44197-024-00262-0.

## Introduction

Excess mortality, defined as the difference between the observed all-cause mortality and the expected one [1, 2], during the COVID-19 pandemic serves as an index for calculating the global illness burden attributed to the emerging SARS-CoV-2 infection [3]. Estimating excess mortality caused by SARS-CoV-2 variants is crucial, as different countries not only experienced different strains and scales of the epidemic [4, 5] but also affected by inadequate medical capacity during the outbreak period of Omicron, one of SARS-CoV-2 variants, after 2021 [6, 7].

In addition to all the factors related to COVID-19 pandemic, all-cause deaths, even before the COVID-19 pandemic, were influenced by factors such as the age structure of the population, Baby Boomers versus non-Baby Boomers, and the capacity of the healthcare system. Therefore, building a background mortality rate model for each country prior to the pandemic is of paramount importance to quantify excess all-cause mortality [8, 9]. It should be highlighted that the aging population, which is responsible for the bulk of COVID-19 and non-COVID-19 deaths, has a substantial effect on excess mortality from all causes, with different countries experiencing distinct patterns of background mortality due to varying health conditions and demographic trends. In Taiwan, the aging Baby Boomer generation has increased background mortality since 2015 [10], impacting medical capacity during large-scale Omicron outbreaks.

From the aspect of methodology, elucidating excess mortality for Baby Boomers and non-Baby Boomers involves time trends, seasonal variations, and calendar year, requiring a time series regression model. Previous models, such as autoregressive time-series and principal component analysis, guided this approach [11–13]. Advanced fuzzy clustering [14] and factor analysis [15] applied to time-series data further classified COVID-19 spread distributions.

Taiwan presents a unique scenario due to the Omicron-naïve Cohort, which refers to the majority of individuals who have not been previously exposed to or infected with other variants of SARS-CoV-2 before Omicron. We sought to compare all-cause mortality during 2020–2022 to the pre-pandemic background mortality from 2015 to 2019. The profiles of excess all-cause mortality across SARS-CoV-2 strains (including Wuhan D614G, Alpha, Delta, and Omicron) were further compared between Baby Boomers (58–76 years as of 2022) and non-Baby Boomers (77  years old and above). Special attention was given to differentiating between direct COVID-19 deaths and indirect non-COVID-19 deaths, particularly from significant community-acquired outbreaks of Omicron VOC. The indirect impacts included strain on medical capacity and healthcare resources, further exacerbating mortality rates during the pandemic.

## Methods

### Study Design and Population

**Fig. 1:**
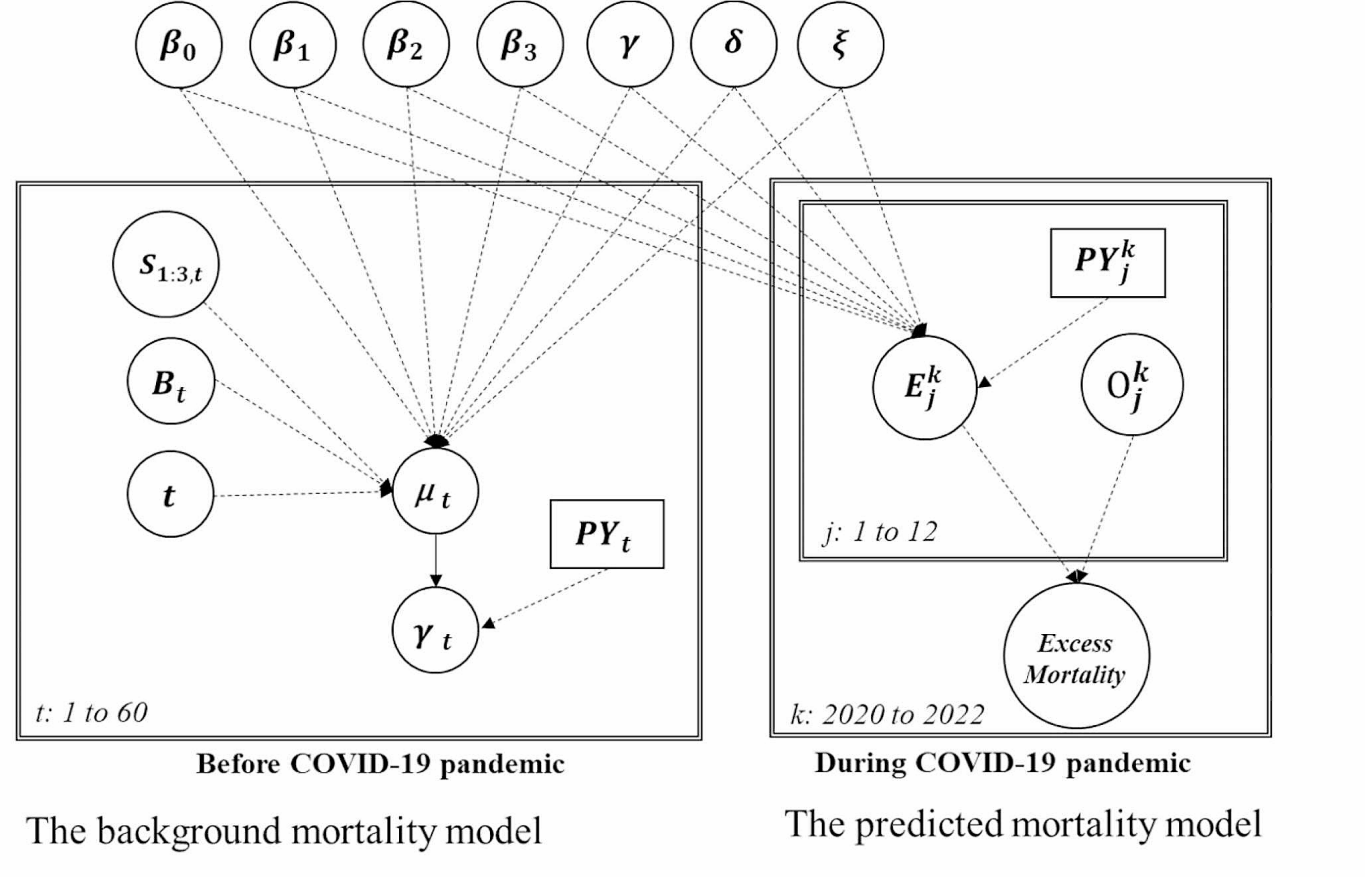
Population-based Poisson time series design study with Bayesian Directed Acyclic Graphic (DAG) approach for evaluation of excess all-cause mortality. $$ {\mu }_{t}$$ and $$ {PY}_{t}$$ represent the monthly average counts and persons years; Regression coefficient presented here include intercept ($$ {\varvec{\beta }}_{0}$$), seasonal variation ($$ {\varvec{\beta }}_{1}-{\varvec{\beta }}_{3}$$), time trend ($$ \varvec{\gamma }\text{ }$$), Baby boomer birth cohort membership ($$ \varvec{\delta }$$), and interaction term ($$ \varvec{\xi }$$)

We used a population-based Poisson time-series design with a directed acyclic graphic (DAG) diagram, as depicted in Fig. 1, to evaluate all-cause excess mortality caused by the outbreak of COVID-19 since 2020. The left panel of DAG is to build the background all-cause mortality model before the COVID-19 pandemic from 2015 to 2019, with the time trend (denoted by t) re-scaled from 1 to 60 months. This background mortality model regresses the counts of all-cause death (denoted by γ_t_) and the corresponding person years (*PY*_t_) at time t on the main independent variable of the birth cohort of Baby boomers (denoted by B), making allowance for seasonal variation (S_1_-S_3_) and time trend (denoted by t), in order to train all the corresponding regression coefficients of each covariate including intercept as shown on the top of Fig. 1. Note that the DAG sorts all relevant variables and regression coefficients into a topological ordering to show a series of causal links with the observed counts of all-cause deaths through the mean counts and person years of the underlying Poisson regression model. The right panel of Fig. 1 then applies the already trained regression coefficients from the background mortality model to building the prediction model for yielding the expected monthly all-cause deaths during the COVID-19 pandemic period from 2020 to 2022 (denoted by k) ($$ {E}_{j}^{k}$$). The ratio of the observed ($$ {O}_{j}^{k}$$) to the expected ($$ {E}_{j}^{k}$$) minus one gives the percentage of excess all-cause deaths resulting from the COVID-19 pandemic. Data from 2015 to 2019 before the COVID-19 pandemic were initially utilized to train the parameters of the background mortality model for obtaining the predicted trend (in dotted yellow) from 2020 to 2022.

We extrapolated the proposed model using Taiwan’s total population of 23,561,236 in 2020, 23,375,314 in 2021, and 23,196,278 in 2022 to forecast the number of deaths that would have happened if there had been no COVID-19 pandemic between 2020 and June 2022 (the dashed line after 2019).

### Data Sources

We utilized all-cause deaths prior to COVID-19 from 2015 to 2019 and during the COVID-19 pandemic period from 2020 to May 2022 [11, 16] (Maleki et al., 2020; Ministry of the Interior, Taiwan, 2022) for calculating excess all-cause deaths in the analysis that follows. We also referred to the reported confirmed COVID-19 cases with two large-scale community-acquired outbreaks, one afflicted by Alpha VOC in May 2021 and the other by Omicron VOC in April 2022, for a better understanding of the corresponding period of each SARS-CoV-2 mutant. (Supplemental Fig. 1)[17]. For death registration, Taiwan's coverage rate is almost 100%. This high level of coverage ensures that mortality data from the Taiwan Viral Registration System is comprehensive and reliable. Beginning in 2015, according to Taiwanese vital statistics, baby boomers accelerated the aging of the population aged 70 and older (Supplementary Fig. 2) [16]. Between 2015 and 2019, the underlying causes of age-related death, which included the vast majority of malignancies and chronic diseases, were responsible for the upward trend of total mortality.

### Statistical Analysis

Following the time series design with DAG as shown in Fig. 1, the background mortality model was developed between 2015 and 2019 by evaluating the regression coefficients of time trend (t), seasonal variation (S_1_-S_3_), Baby Boomer birth cohort membership (B), and the interaction between Baby Boomer birth cohort membership and time trend (B×t). The equation is expressed as follows.

$$log {\mu _t} = \log (P{Y_t}) + {\beta _0} + {\beta _1}{S_1} + {\beta _2}{S_2} + {\beta _3}{S_3} + \gamma t + \delta B + \xi (B \times t)$$

$t=\text{1,2},\dots,60$ months from 2015 to 2019

µ_t_ and PY_t_ represent the average monthly counts and persons years; Regression coefficients presented here include intercept (β_0_), seasonal variation (β_1_-β_3_), time trend (γ), Baby Boomer birth cohort membership (δ), and interaction term (ξ).

Extrapolating the estimated regression coefficients to the COVID-19 pandemic period as shown in the right panel of Fig. 1 would yield the expected all-cause deaths in the absence of the COVID-19 pandemic, which would then be compared to the observed all-cause deaths in the contemporaneous period in order to determine all-cause excess deaths during the COVID-19 pandemic period. The detailed calculation is given as follows,

The expected monthly (j = 1–12) all-cause deaths during the COVID-19 pandemic period (K = 2020–2022) are denoted as $$ {E}_{j}^{k}$$ which is compared with the corresponding observed all-cause death $$ {O}_{j}^{k}$$

Excess all-cause deaths for the kth calendar year $=\sum _{j}({O}_{j}^{k}-{E}_{j}^{k})/{E}_{j}^{k}$  

$$ j:$$ from 1 to 12 months

k: the kth calendar year from 2020 to 2022.

We used Bayesian Markov Chain Monte Carlo simulation (MCMC) procedure for estimating the parameters of regression coefficients, predicting the expected all-cause deaths, and computing the excess all-cause mortality as presented in Fig. 1 [18]. Note that a series of non-informative prior normal distributions N (0, 10^4^) were applied for regression coefficients. The Metropolis Hasting sampling scheme using the random walk model as the proposal distribution was used to yield posterior distribution of each parameter of interest for deriving the mean estimate and 95% credible interval. The software SAS procedure MCMC was used for the implementation of the Bayesian MCMC method.

### Ethical Considerations

Since the datasets included in this investigation were publicly accessible, no ethical review or permission was necessary.

## Results

**Fig. 2:**
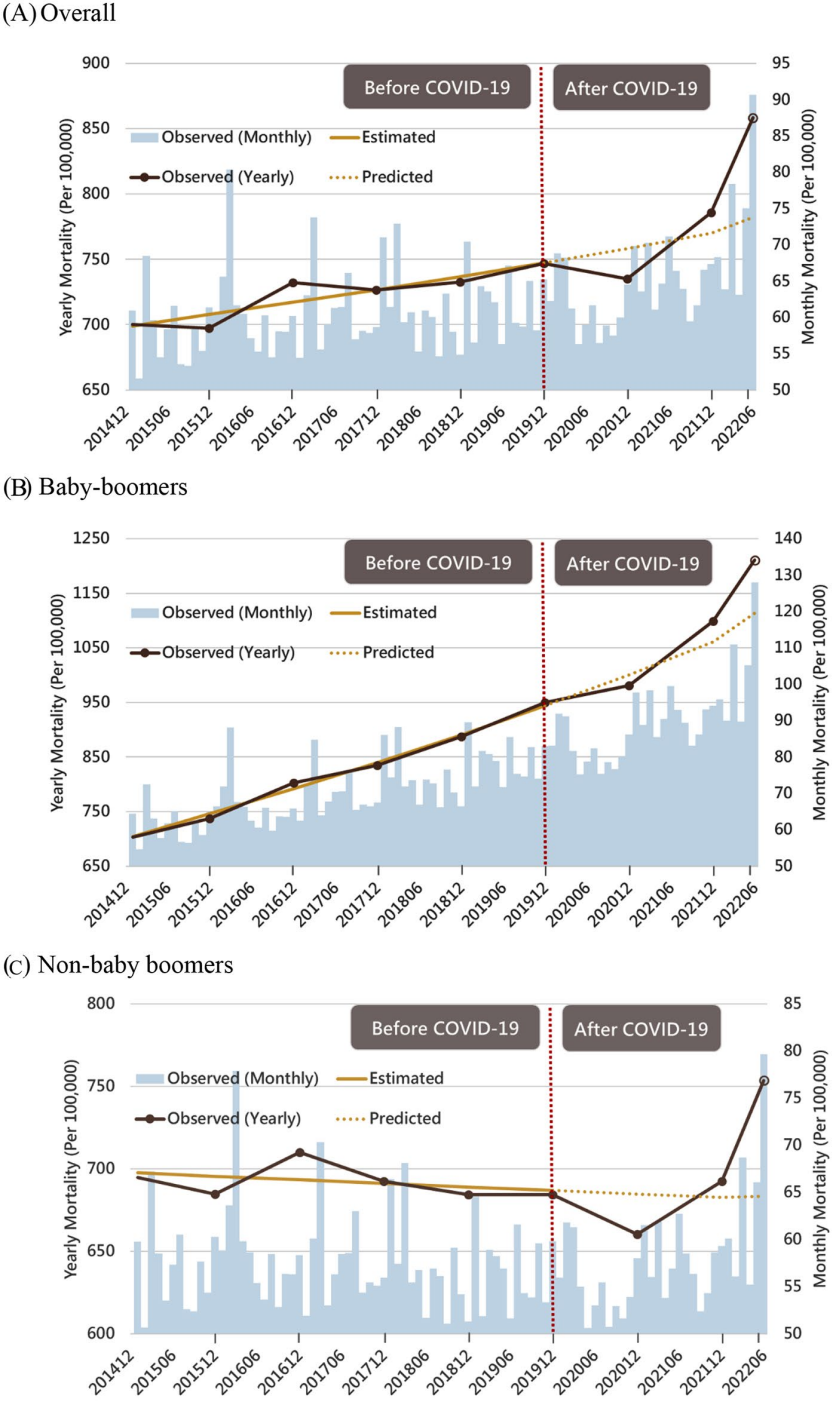
The population-based time series design study to assess excess all-cause deaths for the impact of COVID-19 pandemic among Baby Boomers and non-Baby Boomers in Taiwan, 2015–2022 June

Figure 2(A) illustrates the expected time trend for the total population prior to the COVID-19 pandemic and the predicted time trend for the period following the COVID-19 pandemic. Table 1 depicts the observed number of deaths from all causes from 2020 to June 2022, as well as the projected number of deaths from all causes in the absence of a COVID-19 pandemic, resulting in estimated excess mortality figures. In 2020, when Wuhan D614G COVID-19 was the predominant strain, there were negative excess mortalities (-3%, 95% CI: -4%--3%). This was followed by moderate excess deaths (2%, 95% CI: 1-3%) during the Alpha and Delta VOC periods in 2021 and considerable excess deaths (10%, 95% CI: 9-11%) from January to June during the Omicron pandemic period in 2022. From January to April 2022, when there were modest community-acquired epidemics in northern Taiwan, there were no more deaths. As illustrated in **Supplemental Fig. 1**, there was a considerable rise in mortality in May (18%, 95% CI: 17-19%) and June (43%, 95% CI: 41-44%) as a result of widespread outbreaks of Omicron infection throughout Taiwan’s counties.

**Table 1:** Observed, expected all-cause deaths and estimated excess mortality in Taiwan from 2020 until June 2022

Period	Observed deaths (O)	Expected deaths (E)	O-E	Excess Mortality (O/E-1)
**2020**	173,156	178,690	-5,534	-3% (-4%,-3%)
**2021**	183,732	179,957	3,775	2% (1%,3%)
**2022 Jan-June**	102,177	93,069	9,108	10% (9%,11%)
**2022 Jan-Apr**	63,735	63,628	107	0% (-1%,1%)
**2022 May**	17,409	14,722	2,687	18% (17%,19%)
**2022 June**	21,033	14,735	6,298	43% (41%,44%)

*Taiwan’s population: 23,561,236 in 2020, 23,375,314 in 2021 and 23,186,278 in June, 2022

**Supplemental Table 1** displays the estimated coefficients of background mortality utilized by the Poisson time series regression model to calculate the expected number of deaths from all causes. During 2015–2019, the seasonal fluctuation had a statistically significant impact on the season from January to March compared to other seasons (${{\upchi }}^{2}=$1705.01, *P* <.001). Notable is the statistical significance (${{\upchi }}^{2}=$ 1360.53, *P* <.001) of the interaction between Baby Boomer generation membership and the temporal trend. Both the positive regression coefficients of Baby Boomers and their interaction with the time trend trained prior to COVID-19 suggest that Baby Boomers should anticipate a faster background mortality rate than non-Baby Boomers. According to Supplemental Table 2, the ratio of predicted baseline mortality before and during the COVID-19 pandemic period between Baby Boomers and non-Baby Boomers grew from 1.07 in 2015 to 1.63 in 2022.

Figure 2(B) and (C) demonstrate that Baby Boomers have a different picture of excess mortality from all causes than non-Baby Boomers. Non-Baby Boomers saw 2% more negative excess deaths in 2020 (D614G) and 3% fewer excess deaths in 2021 (Alpha and Delta) than Baby boomers. During the epidemic Omicron period from January to June 2022, excess mortalities from all causes rose to comparable levels for both groups: 9% (95% CI: 7-10%) for Baby Boomers and 10% (95% CI: 9-11%) for non-Baby Boomers (Table 2). In June 2022, when comparing Baby Boomers to non-Baby Boomers divided into two categories < 58 years and > 76 years, excess all-cause deaths in Baby Boomers aged 58–76 years were only marginally lower than excess all-cause deaths in non-Baby Boomers aged 77 years or older. In May 2022, an identical discovery was made.

**Table 2:** Estimated excess mortality in Baby Boomers and non-Baby Boomers in Taiwan from 2020 until June 2022

	Baby boomers	Non-Baby boomers		
	58–76 years	< 58 and > 76 years^a^	> 76 years^b^	< 58 years^b^
**2020**	-2% (-3%,-1%)	-4% (-4%,-3%)	-4% (-4%,-3%)	-5% (-6%,-4%)
**2021**	4% (2%,5%)	1% (1%,2%)	2% (1%,3%)	-1% (-2%,0%)
**2022**	9% (7%,10%)	10% (9%,11%)	8% (7%,9%)	0% (0%,1%)
**2022 Jan-Apr**	0% (-2%,1%)	0% (-1%,1%)	-3% (-3%,-2%)	-6% (-7%,-5%)
**2022 May**	16% (14%,18%)	19% (18%,21%)	19% (18%,20%)	5% (4%,6%)
**2022 June**	40% (37%,42%)	44% (43%,46%)	45% (44%,46%)	23% (21%,24%)

a: Two groups (Baby Boomers and non-Baby Boomers) were modelled in the Poisson time series regression model

b: Three groups (Non-Baby Boomers < 58 years, Baby Boomers (58–76 years), and non-Baby Boomers > 76 years) were in the Poisson time series regression model

Table 3 compares COVID-19 and non-COVID-19 mortality among Baby Boomer and non-Baby Boomer excess all-cause deaths between May and June during the Omicron outbreak. The correlation between Baby Boomers (yes/no) and COVID-19 deaths (yes/no) between May and June was statistically significant (*P* =.005). COVID-19 was responsible for 29% of Baby Boomer mortalities and 33% of non-Baby Boomer deaths. Baby Boomers had a greater proportion of non-COVID-19 deaths than other generations. Non-Baby Boomers should have had a higher incidence of non-COVID-19 than Baby Boomers aged 58 to 76 in 2022, as the latter group’s deaths comprised a considerable number of those aged 77 or older, who would have had a larger need for medical treatment. These data imply that the hastened aging of Baby Boomers has not only been impacted by the Omicron VOC infection, but also by insufficient medical capacity as a result of a lack of health personnel due to restricted containment efforts. Inadequate medical capacity became increasingly apparent in June, as the proportion of non-COVID-deaths jumped from 15% to 17% for non-Baby Boomers and Baby Boomers, respectively, in May to 36% and 40%, respectively, in June.

**Table 3:** The comparison of COVID-19 and non-COVID-19 excess deaths among estimated excess all-cause deaths in Taiwan from 2020 until June 2022

	Excess deaths (%)		*P*-value
	COVID-19	Non COVID-19	
**May-June 2022**			0.005
Baby boomer	1821 (67.0%)	897 (33.0%)	
Non-baby boomer	4427 (70.6%)	1840 (29.4%)	
**May 2022**			0.253
Baby boomer	640 (83.3%)	128 (16.7%)	
Non-baby boomer	1633 (85.1%)	286 (14.9%)	
**June 2022**			0.005
Baby boomer	1181 (60.5%)	769 (39.5%)	
Non-baby boomer	2794 (64.3%)	1554 (35.7%)	

**Fig. 3:**
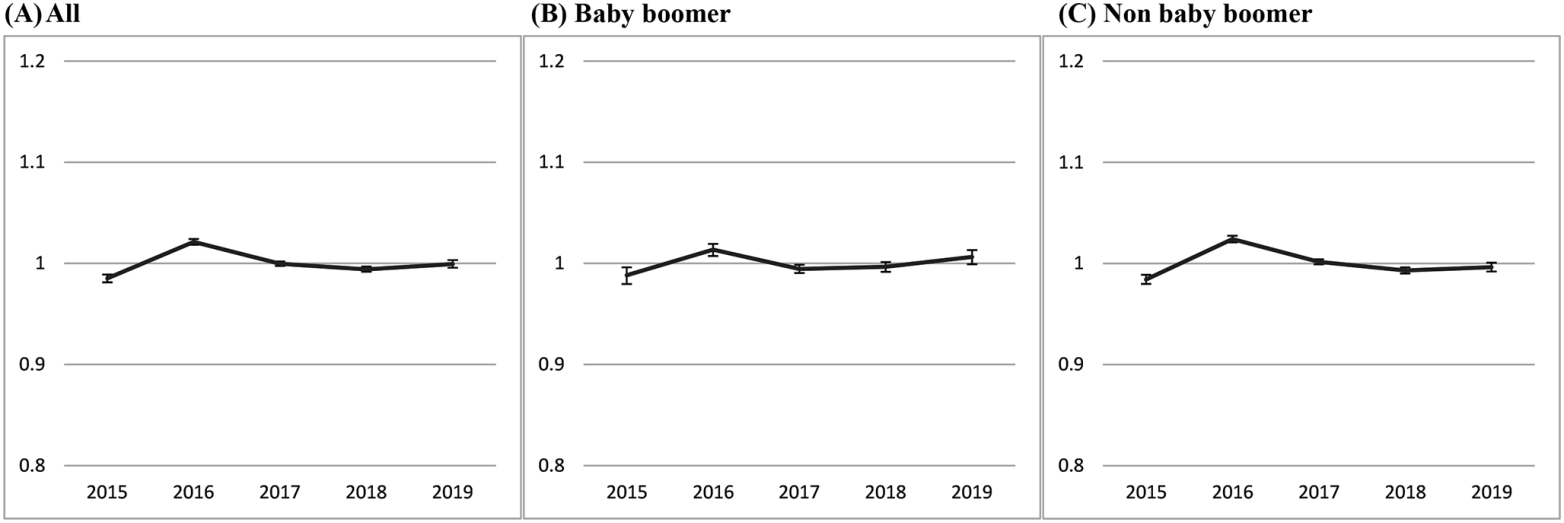
Goodness of Fit (Observed/Expected ratio) for training population-based Poisson time series using Taiwan data from 2015–2019

To check whether the estimated parameters are close to the empirical population-based data before the COVID-19 pandemic period, Fig. 3 shows a plot regarding the ratio of the observed to the expected all-cause deaths estimated from the background mortality between 2015 and 2019. The findings on the ratio close to 1 for each calendar year, particularly the Baby-boomers, revealed the adequacy of the trained model for predicting excess all-cause deaths during the COVID-19 pandemic.

## Discussion

After comparing the reported all-cause deaths during COVID-19 pandemic with the expected all-cause deaths extrapolated from the background mortality before COVID-19 pandemic evaluated by the population-based Poisson time series design with the DAG approach, we found negative excess deaths (-3%) for Wuhan D614G in 2020, 2% excess deaths for Alpha and Delta VOC in 2021, and 10% excess deaths of the Omicron pandemic from January to June in 2022. Compared to non-Baby Boomers (including those aged < 58 years and aged > 76 years), there were 2% fewer negative excess deaths in 2020 with the dominant Wuhan D614G type and 4% more excess deaths in 2021 with the dominant type of both Alpha and Delta SARS-CoV-2 variants among Baby Boomers aged 58–76 years. During the epidemic Omicron period from January to June 2022, the total number of excess deaths grew to nearly identical levels for both groups: 9% for Baby Boomers and 10% for non-Baby Boomers. COVID-19 deaths accounted for 29% and 33% of all-cause deaths among Baby Boomers and non-Baby Boomers, respectively, but Baby Boomers had a higher proportion of non-COVID-19 deaths.

Baby Boomer effects on excess all-cause mortality differ from non-Baby Boomer effects in two respects. As demonstrated in Fig. 2**(B) and (C)**, prior to the COVID-19 pandemic, Baby Boomers had an increasing trend of background mortality, but non-Baby Boomers had a stable and slightly dropping trend. This complicates the estimate of excess mortality from all causes throughout the period of the COVID-19 pandemic. Higher background mortality may be indicative of access to medical care and preventive services in a large, rapidly aging Baby Boomer population prior to the COVID-19 pandemic, and it may also account for different profiles of excess all-cause death in Taiwan between 2020 and 2021 when the epidemic is under control. Thus, excess mortalities from all causes would be predominantly determined by background mortality rather than COVID-19 deaths. In 2020, non-Baby Boomers experienced more negative excess deaths than Baby Boomers. This finding suggests that negative excess deaths may be attributable to the aversion of deaths due to the reduction of influenza-related pneumonia due to masking and personal hygiene or accidents due to reduced social mobility, especially for non-Baby Boomers aged 77 or older in 2020 when Taiwan had a very strict containment policy [19, 20]. However, compared to non-Baby Boomers, Baby Boomers aged 58–76 years had fewer negative excess deaths in 2020 and more excess deaths in 2021 because the indirect benefit of strict containment measures may be offset by the Baby Boomers’ already low medical utilization for their high need for medical care, which could worsen if strict containment measures were in place during the COVID-19 pandemic. Second, when both Baby Boomers and non-Baby Boomers experienced a community-acquired outbreak of Omicron VOC between May and June 2022, they experienced the same significant excess deaths, supporting the previous finding that excess deaths were more prominent for Omicron VOC outbreaks than Delta VOC outbreaks [21]. Nonetheless, the profiles of Baby Boomers and non-Baby Boomers who are responsible for an excess of mortalities from all causes remain distinct. Non-Baby Boomers may see an increase in mortality, particularly among the elderly aged 80, due to the loss of indirect benefits between 2020 and 2021, as well as the high likelihood of Omicron infection due to the disproportionately high proportion of unvaccinated older individuals. On the other side, an increase in non-COVID-19 deaths due to a scarcity of health-care personnel may be associated with an increase in total Baby Boomer mortality. Although the direct cause of case fatality for Omicron was lower than for other SARS-CoV-2 variant like Delta, the indirect reason of a scarcity of health workers due to the high Omicron infection could have a higher impact during the pandemic era of Omicron. The WHO report [6] promotes direct and indirect influences strongly.

The evaluation of excess mortality also plays a significant role in determining the global illness burden when the issue of underreporting in particular countries is recognized. Wang et al. (COVID-19 Excess Mortality Collaborators, 2022) employed an effective statistical model to estimate the magnitudes of excess mortality during the COVID-19 pandemic period from the beginning of 2020 to the end of 2021 by country’s level of economic development. After fitting the proposed model, which accounted for a multitude of significant factors, they found that the reported number of mortalities from all causes worldwide was underestimated by one-third. Later, the WHO developed a revised form of excess mortality, concentrating only on Germany and Sweden [3]. One of the reasons for the correction is to determine whether or not it is appropriate to utilize mortality rates from before the COVID-19 epidemic. In contrast to these valuable studies that focused on estimating the global disease burden across countries, we evaluated excess mortality at the country level for those affected by aging Baby Boomers with an increasing background mortality trend prior to the COVID-19 pandemic period in order to reflect an unbiased estimate of excess all-cause deaths during the COVID-19 pandemic period. Supplemental Fig. 3 depicts countries with and without a Baby Boom. These results demonstrate conclusively that the Baby Boom had a far bigger impact on excess mortality during the Omicron VOC pandemic phase than prior SARS-CoV-2 strains. The findings also highlight the importance of exercising caution when interpreting excess all-cause deaths based on global comparisons when evaluating excess all-cause mortality at the country level, due to the fact that the background mortality of each country is affected by various factors, such as the Baby Boom effect proposed in this study and also the disparity of health care system across geographic areas and various ethnic groups. However, as the health care system in Taiwan is very homogeneous under a nationwide health insurance system, we still think the underlying disease burden of Baby-Boomers in the face of Omicron, given the reduced medical capacity, would be a strong explanation for excess all-cause mortality on this occasion.

Despite these findings on Baby Boomer-caused excess mortality, our study is limited in elucidating the detailed causes responsible for the disparity of excess all-cause-deaths across different SARS-CoV-2 variants between Baby Boomers and non-Baby Boomers, as the influences of Baby Boomers on excess mortality were intertwined with their diverse characteristics, such as the emerging SARS-CoV-2 subvariants, the compliance with containment measures, susceptibility to COVID-19 infection, the coverage rate of vaccination, the accessibility and the availability of anti-viral therapy, and the resilience to insufficient medical capacity. Additionally, future research should consider how vaccination rates are associated with mortality rates among Baby Boomers and non-Baby Boomers, as this was not addressed in the current study. Therefore, an ongoing research is needed to investigate these factors by performing large retrospective cohort research with the collection of individual-level information in the future. Regarding the emerging SARS-CoV-2 subvariants, it is still important to continuously apply the proposed Poisson time series design with the DAG approach to a series of the emerging SARS-CoV-2 subvariants with higher transmission and evasion of immunity,, such as XBB.1.5 and BQ.1.1, circulating in the USA and Europe as of the writing of this manuscript because monitoring excess all-cause mortality in each period of the emerging subvariant would provide an indicator for the surveillance of the severity of COVID-19 and the provision of medical capacity. In conclusion, the profiles of excess mortality from all causes for Baby Boomers and non-Baby Boomers differed both before and throughout the COVID-19 pandemic era. Prior to the COVID-19 pandemic period, the background mortality rate for Baby Boomers was growing, whereas the trend for non-Baby Boomers was practically stable. In 2020 and 2021, the COVID-19 epidemic caused a rise in the number of Baby Boomer deaths relative to non-Baby Boomer deaths. During the Omicron epidemic period, Baby Boomers aged 58 to 76 had the same excess of all-cause fatalities as non-Baby Boomers aged 77 or older, but they were more likely to die from causes other than COVID-19.

In Taiwan, the fast-aging Baby Boomer generation experienced a high number of deaths from all causes. This could be attributed to a lack of access to medical care when the COVID-19 epidemic is under control from Wuhan D614G, Alpha, and Delta VOC and a lack of medical capacity when dealing with large-scale outbreaks of Omicron VOC.

## Electronic Supplementary Material

Below is the link to the electronic supplementary material.

Supplementary Material 1

## Data Availability

The data sets generated during and/or analyzed during the current study are available from the corresponding author on reasonable request.

## Abbreviations

DAG: directed acyclic graphic
MCMC: Markov Chain Monte Carlo simulation
PY: Person years
SARS-CoV-2: Severe acute respiratory syndrome coronavirus 2
VOC: Variant of Concern
