# Supplementary material for: Assessing Excess Mortality of Baby Boomers from the COVID-19 Pandemic: Taiwan Omicron-naïve Cohort
Source: J Epidemiol Glob Health. 2024 Jun 20;14(3):1113–21. doi: 10.1007/s44197-024-00262-0 (PMC11444035; doi:10.1007/s44197-024-00262-0)
Supplement: Supplementary file 1 — Supplementary Material 1 [file 44197_2024_262_MOESM1_ESM.docx]

**Supplementary Figure 1.** Daily reported number of domestic cases and death before and after large-scale community-acquired outbreaks of Omicron infection in Taiwan


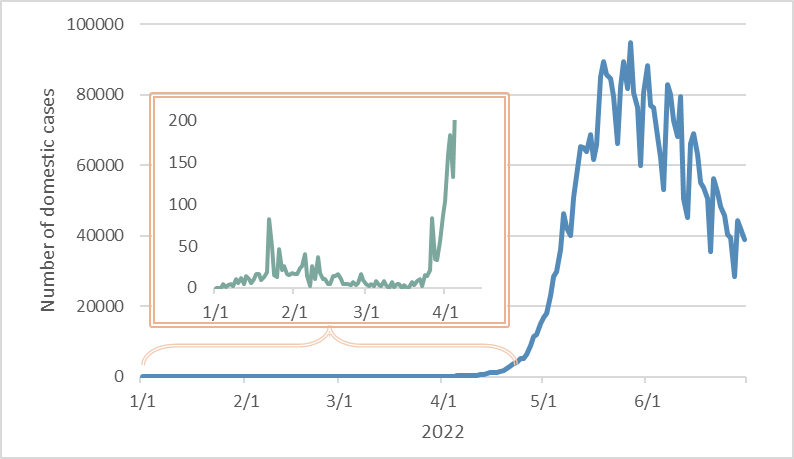
**Supplementary Figure 2.** Time trend of proportion of aged 70 years and above from 1993 to 2021 in Taiwan.

**Supplementary Figure 3.** Estimated excess deaths for COVID-19 pandemic of Asian and Oceania countries

1. **Countries with baby-boom effect**

**
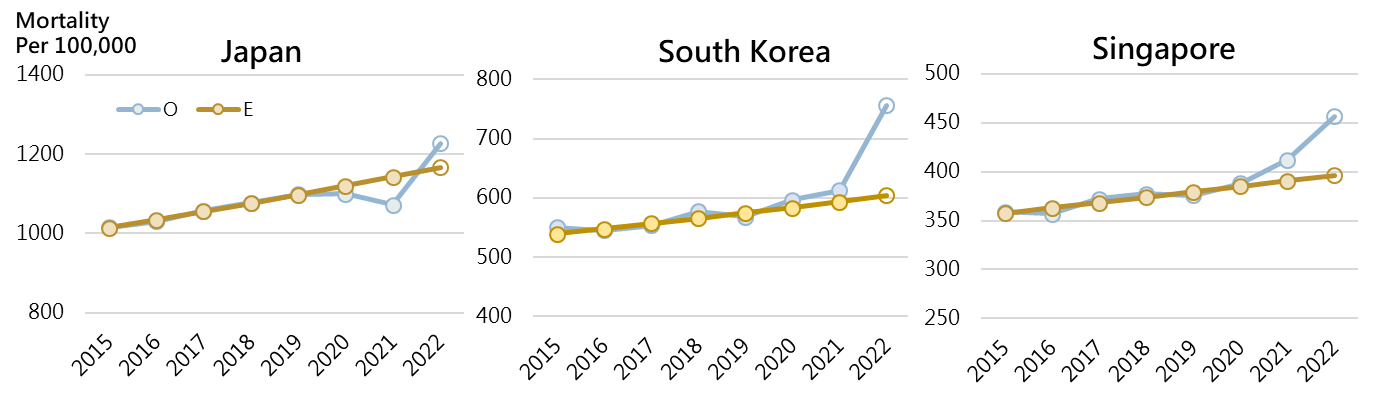
**

1. **Countries without baby-boom effect**

**
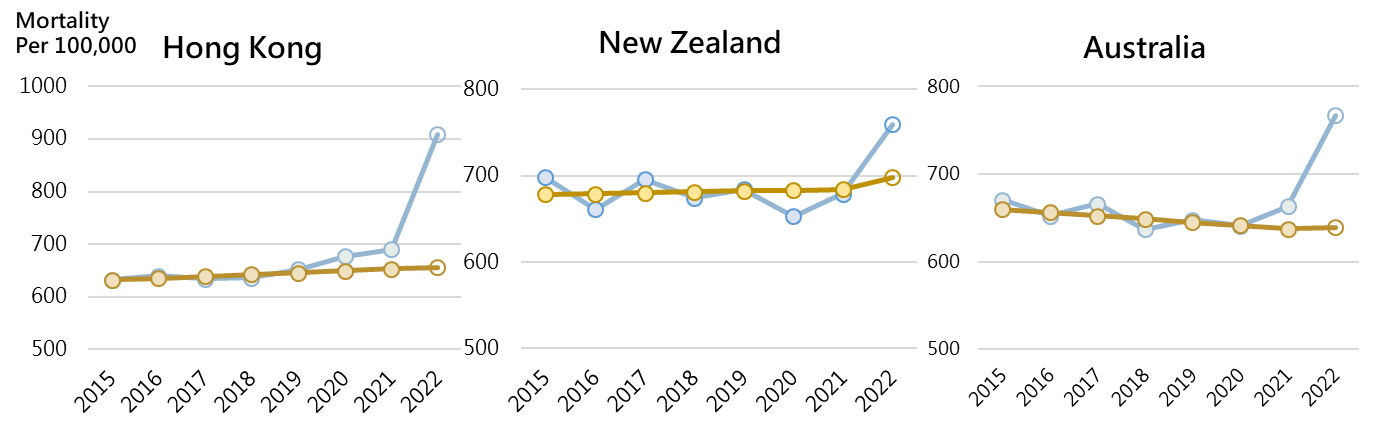
**

**Supplementary Table 1.** Estimated regression coefficients of Poisson time series regression model of background mortality

|  | **Coefficient** | **Standard Error** | **95%CI** | | **P-value** |
| --- | --- | --- | --- | --- | --- |
| **Intercept** | -7.4768 | 0.0034 | -7.4835 | -7.4701 | <.0001 |
| **Trend** |  |  |  |  |  |
| **Per month** | -0.0003 | 0.0001 | -0.0004 | -0.0001 | 0.0006 |
| **Season** |  |  |  |  | <.0001 |
| **(vs. Oct-Dec)** |  |  |  |  |  |
| **Jan-Mar** | 0.1011 | 0.0031 | 0.0951 | 0.1071 |  |
| **Apr-Jun** | 0.0001 | 0.0031 | -0.006 | 0.0062 |  |
| **Jul-Sep** | -0.0046 | 0.0031 | -0.0107 | 0.0015 |  |
| **Baby boomers vs. Non-baby boomers** | 0.0379 | 0.005 | 0.0281 | 0.0476 | <.0001 |
| **Baby-boomer*Trend** | 0.0051 | 0.0001 | 0.0049 | 0.0054 | <.0001 |

**Supplementary Table 2.** Rate ratio of expected mortality by baby-boomers and non-baby boomers

|  | **Baby boomers** | | **Non-baby boomers** | | **Ratio (Baby boomers vs. Non-baby boomers)** |
| --- | --- | --- | --- | --- | --- |
|  | **Number** | **Expected**  **mortality*** | **Number** | **Expected**  **mortality*** |  |
| **2015** | 5,735,026 | 746.49 | 17,757,048 | 695.45 | 1.07 (1.06-1.08) |
| **2016** | 5,691,951 | 791.57 | 17,847,865 | 693.31 | 1.14 (1.14-1.15) |
| **2017** | 5,647,807 | 839.37 | 17,923,420 | 691.18 | 1.21 (1.21-1.22) |
| **2018** | 5,600,697 | 890.05 | 17,988,235 | 689.05 | 1.29 (1.29-1.30) |
| **2019** | 5,553,130 | 943.79 | 18,049,991 | 686.93 | 1.37 (1.36-1.38) |
| **2020** | 5,487,530 | 1000.78 | 18,073,706 | 684.82 | 1.46 (1.45-1.48) |
| **2021** | 5,382,096 | 1061.21 | 17,993,218 | 682.71 | 1.56 (1.54-1.57) |
| **2022** | 5,307,761 | 1113.49 | 17,878,517 | 683.46 | 1.63 (1.60-1.65) |

***Per 100,000**
